# Analysis of the emotional experiences of Japanese Alcoholics Anonymous members striving for sobriety

**DOI:** 10.1186/s12888-019-2226-0

**Published:** 2019-08-06

**Authors:** Miyuki Kihara, Kazuyo Kitaoka

**Affiliations:** 1 Department of Health Sciences, Faculty of Medical Sciences, Kyushu University, 3-1-1 Maidashi Higashi-ku, Fukuoka, 812-8581 Japan; 2 Department of Nursing, Faculty of Health Sciences, Komatsu University, Nu 1-3, Schichomachi, Komatsu, Ishikawa 923-8511 Japan

**Keywords:** Alcoholism, Alcohol use disorder, Alcoholics Anonymous, 12-step programs, Abstinence, Emotions, KJ method, Recovery

## Abstract

**Background:**

Japan’s tolerance of alcohol consumption and intoxication pose extreme difficulties for community-dwelling alcoholics who wish to abstain from alcohol and maintain sobriety. Emotional problems triggered in daily life can easily lead to relapse, especially after abstinence. Alcoholics Anonymous places great emphasis on dealing with attendees’ emotional experiences. However, the specific nature of this support is not well understood. Therefore, this study aimed to elucidate the emotional experiences of AA members who strive for sobriety while attending AA and to identify suggestions for new methods of support.

**Methods:**

The present study employed an interview method. Data were analyzed using the KJ Method, which was developed by Japanese cultural anthropologist Jiro Kawakita. Study participants were 36 men chosen from AA groups in the Kanto and Kyushu regions of Japan.

**Results:**

Long-term abstinence was achieved through the following process: 1) gaining objectivity, 2) striving to maintain an attitude of acceptance, and 3) remaining devoted to discipline for one’s goals, thereby 4) recovering one’s contradictory self. This was an unending process that unfolded as the individual tasks affected each other. To facilitate this process, AA members dealt with risk cues that destabilized post-cessation emotional balance while making daily efforts to regulate their own emotions.

**Conclusions:**

To maintain sobriety, AA members strove to regulate their emotions. By doing so, they experienced personal growth and attained a life in which they did not require alcohol (i.e., sobriety). The present study indicated that Japanese nursing and health care workers should be willing to learn from AA members, such as by understanding the 12-step culture. The data also suggest the need to create environments conducive to AA activities, from which many alcoholics derive emotional support.

## Background

Alcohol use disorder is a significant global health challenge. During the 2010 World Health Assembly, the World Health Organization adopted a Global Strategy to Reduce Harmful Use of Alcohol. Seizing this opportunity, the Japanese Society of Alcohol-Related Problems and other groups concerned with alcohol use disorder undertook efforts, which resulted in the passage of the Basic Act on Measures against Alcohol-related Harm (Act No. 109 of 2013) in December 2013.

In studies that ranked only the harms of 20 listed drugs, heroin and crack cocaine were positioned as the most harmful to users. However, in a comprehensive review that also took into account the harm to society, alcohol was reported to be even more harmful than heroin or crack cocaine [1]. Another study reported that there was no optimally safe level of alcohol consumption, and that alcohol use contributed to loss of health resulting from various causes [2]. The true impact of alcohol on people’s health is still being debated from a global perspective.

In contrast, while Japan has long advocated responsible alcohol consumption [3], Japanese society is generally tolerant of drinking [4]. Drunkenness tends to be approached sympathetically, and alcohol disorder use has not historically been viewed as a major social problem [5]. In 2012, among the 1.09 million Japanese individuals diagnosed with alcohol use disorder according to International Classification of Diseases and Related Health Problems 10th Revision criteria, only an estimated 80,000 were undergoing treatment—a finding that indicates that the vast majority of alcoholics in Japan are not receiving the treatment they need [6]. Drunk driving, which had been disregarded for decades, has finally come to be recognized as a social problem in recent years due to a seemingly endless series of accidents caused by drunk drivers. Other issues, such as the emergence of drinking problems following natural disasters, have drawn the attention of the Japanese populace toward alcohol-related problems. Meanwhile, the media demonstrate a fundamental misunderstanding of drinking problems among celebrities and tend to blame the individual for his or her drinking problem. Moreover, modern Japanese people still believe in part that “saké is the best medicine.” These factors combine to make it difficult for Japanese people to gain a clear understanding of the nature of alcohol use disorder.

Alcohol use disorder is a frightening disease and once a person develops it, they cannot solve the problem simply by trying to avoid alcohol or resolving not to drink it. They must stop living their old life, in which alcohol is an object of fixation, and construct a new life in which they do not drink alcohol. The diversity of personal experiences among alcoholics makes it difficult to find clues to aid in recovery [7]. In addition to the temporary change in emotions following recovery from alcohol use disorder, emotional experiences triggered in daily life are especially likely to lead to relapse, meaning that dealing with emotional aspects is crucial for effective treatment [8]. In such circumstances, the mutual-help group Alcoholics Anonymous (hereafter “AA”) is a valuable resource for community-dwelling alcoholics in Japan.

AA members from foreign countries have been engaged in activities in Japan since 1952. Some of these members also spoke at Japanese hospitals. In the 1960s, AA groups were established at US military bases in Yokohama, Okinawa, Kadena, Yokosuka, and Sasebo. At that time, meetings were held in English. In 1974, an alcoholic American priest in Tokyo provided a helping hand to an alcoholic Japanese priest, and since then AA activities have been actively conducted in the capital. The first step-meeting conducted in Japanese was held on March 16, 1975 in Kamata, Tokyo. This can be viewed as the beginning of AA in Japan because it began the practice of steps, the AA program of recovery [9]. AA events were subsequently held across Japan. After officially registering as the “Tokyo Group” with the New York General Service Office in 1976, the activities became more focused and AA groups started across Japan. The AA Japan General Service Office was established in 1981 and was registered as the “NPO corporation AA Japan General Service” in April 2005. Its seven central offices continue to play an important role as a social resource in Japan [10].

The AA program places great emphasis on dealing with members’ emotional experiences [11–13]. However, the specific nature of this support and the experiences of attendees have not been adequately examined in Japan. Although studies have been conducted in the United States on AA’s 12 steps and the mechanisms of change for AA members attributable to spirituality [14, 15], no such studies have been conducted in Japan. We felt that knowing what kind of emotional experiences Japanese people receive from AA participation may be useful in rethinking the nature of support for abstaining alcoholics. This study therefore had two major aims. First, we sought to understand the emotional experiences of alcoholics who strive for sobriety while attending AA. Second, we wanted to examine the emotional processes related to AA experiences.

Neither AA attendees themselves nor medical experts can predict who will be able to maintain abstinence. Despite this perplexity, the present study is one of relatively few to have directly interviewed AA members who have maintained sobriety. The participants in this study are among the few people who continue to diligently face their disease in a mutual-help setting. The experiences of these people provide a vital opportunity to present the long-term prospects of alcoholics not only to nurses and other health care workers, but also to alcoholics and their families.

## Methods

### Study design

An interview method was used. Data were collected via interviews, which followed a semi-structured format according to an interview guide created for this study (Appendix). Interviews were recorded with a digital voice recorder, for which consent was obtained from participants. After the interview, the recordings were transcribed verbatim and were then analyzed.

This study deals with emotions, which are habitual and complex experiences. Hence, consistent with the idea that richly nuanced qualitative data are more appropriate than numerical data, the present study was conducted using a qualitative design.

### Ethical considerations

This study was approved by the Ethics Committee of the Kanazawa University School of Medicine (approval No. 185). Written consent was provided by participants following receipt of written and oral explanations from the researchers. Participants’ personal information was protected, and participants were assured that refusal to participate would not bring any disadvantage; that all information obtained would not be used for any purpose not related to the study; and that participants were free to withdraw consent or discontinue participation at any time, and that doing so would not bring any disadvantage.

### Participants

Study participants were 36 men who attended AA and had been identified as likely to have alcohol use disorder using an alcohol use disorder screening test with the Diagnostic and Statistical Manual of Mental Disorders, Fifth Edition (DSM-5) diagnostic criteria [16] serving a supplemental role. Because women are mainly responsible for housework and childcare in Japan and are thought to have different emotional experiences than men, we focused on male experiences in this study. Participants were recruited in the Kanto and Kyushu regions from current AA members, of whom there are an estimated 4800 nationwide [17]. Participants were recruited via a written explanation of the study given to them by the leaders of their AA chapters. Of the 36 participants, 18 were in their thirties to sixties (mean age, 53.06 years). The participants had maintained abstinence from alcohol for a mean duration of 13.76 (range, 1–35) years.

### Data collection

In a preliminary investigation with three study participants aimed at confirming the interview items, the participants were sometimes at a loss for words to accurately express their emotions regarding alcohol. Therefore, to help them put their emotions into words, we created a list of emotions for use during interviews.

### Creation of emotion list

Emotions are subjective experiences which involve ambiguity and are thus difficult to distinguish, even for the person directly experiencing them. Therefore, we examined types of emotions via three components: the list of “Emotion Names” developed by Hochschild [18], which is based on Ekman’s basic emotions of happiness, surprise, anger, fear, disgust, and sadness [19, 20]; Miyamoto’s “Components of Sense of Incongruity” [21]; and cases in which a similar method was used to explore emotions [22]. Using this method, we produced a list of 36 emotions to help distinguish emotions (Table 1). During interviews, this emotion list was posted in front of participants to serve as an aid as they spoke not only about their experiences, but also the emotions experienced. The aim was to make it easier for participants to express the emotions they felt.

**Table 1:** List of emotions

Happiness Exhilaration Relief Sadness Surprise Fear Anger	
Revulsion Shame Contempt Jealousy Envy Guilt	
Irritation Disappointment Resentment Doubt Suspicion Betrayal	
Helplessness Pity Self-reproach Loneliness Melancholy Anxiety	
Frustration Depression Impatience Inadequacy Humiliation Misery	
Shame Dread Excitement Abandonment Sympathy	

### Analysis

Data recorded during interviews were transcribed verbatim. The cultural anthropologist, Jiro Kawakita, developed the KJ Method to draw out the essence of the data with inspiration from the data itself. The KJ method eliminates the subjectivity of the analyst and lets the data speak for themselves.

As an exploration of data organization, the KJ method in its narrowest sense is perhaps similar to thematic analysis. However, in its broader sense, the KJ method encompasses everything from methods of data collection and organization to problem solving [23] and it goes beyond the simple classification undertaken by thematic analysis. The KJ method classifies data while emphasizing finding similarities in the data, which may seem to be different things at first glance. Then, ideas are intuitively created by integrating data qualitatively and illustrating it as a diagram. We see it as a series of “thinking methods” that activate the sensitivity needed to try to capture the essence of things. Moreover, we think that its difference from thematic analysis also comes out in the form of the affinity diagrams.

We analyzed and structured data as follows. 1) Creation of labels: Verbatim transcripts were carefully read. For each event related to an emotional experience that led to a desire to drink alcohol, we created a label to indicate this experience. 2) Creation of groups: Similar labels were grouped together by duration of abstinence, and we checked whether labels within groups indeed resembled each other. 3) Making nameplates: We abstracted the core concept of all labels gathered into a single group and made a nameplate. Completion of nameplates for all groups constituted the end of one level. The process of grouping and creating nameplates was repeated across several levels until there were ultimately no more than 10 or so nameplates. 4) Spatial arrangement: We compared nameplates, searched for configurations of interrelationships that were easy to understand, and arranged the nameplates. 5) Affinity diagram: The locations of labels were fixed, encircled like islands, and arranged at each level. Interrelationships between islands were then examined to draw an overall relationship diagram [24].

### Ensuring analysis validity

The researchers were trained by Jiro Kawakita, who developed the KJ Method, and Kimiko Kawakita, who taught the KJ Method. In addition, along with other researchers who had received the same training, the researchers participated in KJ Method interactive networking events and in the Society of KJ Method events to accumulate further experience in research using the KJ Method. To reduce the researchers’ own bias in interpretation, a supervisor oversaw the entire study and analysis process. Interim reports were also made to graduate students, researchers engaged in qualitative research, and other researchers using the KJ Method to solicit opinions and suggestions regarding the nature and analysis of the data.

## Results

**Fig. 1:**
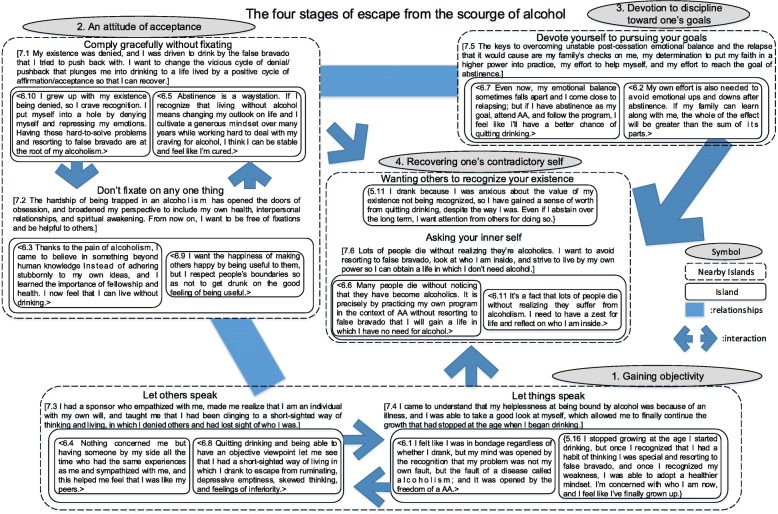
Structure of emotional experiences of Alcoholics Anonymous members in Japan striving for sobriety

Using the KJ method, transcripts were labeled, and carefully selected labels were integrated into seven islands. Verbatim transcripts were read carefully, and 470 passages relevant to the theme of the study were labeled and arranged to create an affinity diagram. Thus, there were a total of 470 labels. Grouping of related labels and creation of nameplates across seven levels yielded seven islands. Furthermore, a schematic representation of the spatial arrangement of the seven islands revealed four “Nearby Islands,” which we then expressed as symbols. This schematic constituted an affinity diagram in which the emotional experiences of Japanese Alcoholics Anonymous members working toward sobriety are characterized by an iterative process of interrelations among these four symbols. Related islands are surrounded by lines, symbols (Nearby Islands) related to each other are connected by lines, and relationships in which islands and symbols affect each other are represented by arrows (Fig. 1). Labels on levels 5, 6, and 7 are indicated by {}, < >, and [], respectively. The storyline for each symbol is described below.

### Gaining objectivity

#### Let others speak

When study participants drank: < 6.4 Nothing concerned me but having someone by my side all the time who had the same experiences as me and sympathized with me, and this helped me feel that I was like my peers.>. < 6.8 Quitting drinking and being able to have an objective viewpoint let me see that I had a short-sighted way of living in which I drank to escape from ruminating, depressive emptiness, skewed thinking, and feelings of inferiority>. This led to the idea that [7.3 I had a sponsor who empathized with me, made me realize that I am an individual with my own will, and taught me that I had been clinging to a shortsighted way of thinking and living in which I denied others and had lost sight of who I was.]. In other words, the participants, when they were drinking, were unable to accept the reality that the way they were drinking was both a nuisance to others and deleterious to their own health; and though this was a matter that pertained to them personally, they felt as though it were happening to someone else. This aspect of themselves became self-evident in the mirror held up by their peers, and helped them to better understand themselves.

#### Let things speak

The participants originally felt trapped. < 6.1 I felt like I was in bondage regardless of whether I drank, but my mind was opened by the recognition that my problem was not my own fault, but the fault of a disease called alcoholism; and it was opened by the freedom of AA.>. By continuing to implement the AA program in fellowship with their peers in AA, things gradually changed: {5. 16 I stopped growing at the age I started drinking, but once I recognized that I had a habit of thinking I was special and resorting to false bravado, and once I recognized my weakness, I was able to adopt a healthier mindset. I’m concerned with who I am now, and I feel like I’ve finally grown up.}. Although participants had consumed alcohol to escape from reality, they began to be bound by the act of alcohol consumption itself without realizing it: [7.4 I came to understand that my helplessness at being bound by alcohol was because of an illness, and I was able to take a good look at myself, which allowed me to finally continue the growth that had stopped at the age when I began drinking].

The ideas of “letting others speak” and “letting things speak” seemed to mutually influence each other and contributed to emotional processes conducive to involvement with AA and supporting sustainable recovery.

#### An attitude of acceptance

##### Comply gracefully without fixating

At some point, participants started trying to quit drinking after coming to a realization: < 6.10 I grew up with my existence being denied, so I crave recognition. I put myself into a hole by denying myself and repressing my emotions. Having these hard-to-solve problems and resorting to false bravado are at the root of my alcoholism.>. But it was only through mental resolve that they were able to accept their situation: < 6.5 Abstinence is a waystation. If I recognize that living without alcohol means changing my outlook on life and I cultivate a generous mindset over many years while working hard to deal with my craving for alcohol, I think I can be stable and feel like I’m cured. > Because they always felt that their existence had been denied, it became a habit to force a kind of false bravado, and that forced behavior seemed to be linked with their drinking. Transforming a stubborn attitude in which, even if they were in the wrong, they would not admit their error but, on the contrary, become hostile, and adopting a more composed attitude seems to hold the key to solving the drinking problem. In other words, they arrived at the idea that [7.1 My existence was denied, and I was driven to drink by the false bravado that I tried to push back with. I want to change the vicious cycle of denial/pushback that plunges me into drinking to a life lived by a positive cycle of affirmation/acceptance so that I can recover].

##### Don’t fixate on any one thing

Participants meekly accepted the pain of giving up alcohol and awoke to a kind of spirituality: < 6.3 Thanks to the pain of alcoholism, I came to believe in something beyond human knowledge instead of adhering stubbornly to my own ideas, and I learned the importance of fellowship and health. I now feel that I can live without drinking. > In order to keep holding on to this feeling without ever losing it, participants exercised caution against unexpected pitfalls, even after remaining sober: < 6.9 I want the happiness of making others happy by being useful to them, but I respect people’s boundaries so as not to get drunk on the good feeling of being useful.>.

In other words, they arrived that the idea that [7.2 The hardship of being trapped in alcoholism has opened the doors of obsession and broadened my perspective to include my own health, interpersonal relationships, and spiritual awakening. From now on, I want to be free of fixations and be helpful to others.].

To “comply gracefully without fixating” and “not to fixate on any one thing” also exercised a mutual influence on one another, and as long as participants were able to maintain these attitudes, these approaches seemed to contribute to the emotional processes conducive to involvement with AA and supporting sustainable recovery.

#### Devotion to discipline toward one’s goals

##### Devote yourself to pursuing your goals

< 6.7 Even now, my emotional balance sometimes falls apart and I come close to relapsing; but if I have abstinence as my goal, attend AA, and follow the program, I feel like I’ll have a better chance of quitting drinking. > In other words, even if the participant has been abstinent for a long time, some event could still disrupt the emotional balance and lead to the temptation to drink. Being able to remain sober for a long time was also described as simply becoming proficient, with the help of their peers at AA, at overcoming the dangers inherent in relapsing into drinking. < 6.2 My own effort is also needed to avoid emotional ups and downs after abstinence. If my family can learn along with me, the whole of the effect will be greater than the sum of its parts. > Thus, participants also understood and practiced the importance of putting in place a surrounding environment conducive to continued sobriety. [7.5 The keys to overcoming unstable post-cessation emotional balance and the relapse that it would cause are my family’s checks on me, my determination to put my faith in a higher power into practice, my effort to help myself, and my effort to reach the goal of abstinence.] Thus, in their reliance on AA fellowship and a higher power, the individuals themselves will also play a key role in how they maintain their efforts to continue on the AA program and maintain their own sobriety. To be able to devote oneself to pursuing one’s own goals seemed to contribute to the emotional processes conducive to involvement with AA and supporting sustainable recovery.

## Recovering one’s contradictory self

### Asking your inner self

Here, “contradictory self” refers to the emotional conflict in which continued sobriety is impossible without abandoning the narcissistic elements characteristic of abstaining alcoholics and without acquiring objectivity and maturity [25]. < 6.11 It’s a fact that lots of people die without realizing they suffer from alcoholism. I need to have a zest for life and to reflect on who I am inside. > Thus, faced with the reality of the frequent death of their AA peers, participants exercised humility in practicing their own AA program while reflecting on their inner selves on a daily basis. Then, participants were filled with a feeling of gratitude: < 6.6 Many people die without noticing that they have become alcoholics. It is precisely by practicing my own program in the context of AA without resorting to false bravado that I will gain a life in which I have no need for alcohol.>. [7.6 Lots of people die without realizing they’re alcoholics. I want to avoid resorting to false bravado, look at who I am inside, and strive to live by my own power so I can obtain a life in which I don’t need alcohol.] In this way, they felt that no matter what happened, they would be able to remain free of the need for alcohol precisely by continuing to steadily practice the AA program without resorting to false bravado.

### Wanting others to recognize your existence

On the other hand, some were unable to stop drinking much due, in large part, to their desire to have the value of their own existence recognized. {5.11 I drank because I was anxious about the value of my existence not being recognized, so I have gained a sense of worth from quitting drinking, despite the way I was. Even if I abstain over the long term, I want attention from others for doing so.} In this way, because they felt they have never been acknowledged by others (e.g., their parents), they felt pride in the very fact that they continued to live a sober life. They were also aware of the dangers of their tendency to desire and seek attention. Attention and strong emotions quickly led to drunkenness and were dangerous in that they might lead to a thirst for alcohol. They were aware that continuing to practice the program in fellowship with their AA peers on a daily basis was an essential task in maintaining their sobriety.

From the above, as long as one continues “asking one’s inner self,” this would seem to contribute to the emotional processes conducive to involvement with AA and supporting sustainable recovery. However, if one neglects to “ask one’s inner self” and “the desire for others to recognize your existence” grows stronger, this would seem to be a factor inhibiting the promotion of emotional processes conducive to such involvement with AA and supporting sustainable recovery. It was considered that “Recovering one’s contradictory self” would continue to be an ongoing task into the future for maintaining sobriety on the part of participants.

## Discussion

The participants in this study were members of AA. Therefore, we shall discuss the results of the present study in terms of their relationship to the AA program and fellowship through AA.

### Characteristics of emotional experiences of AA members in Japan striving for sobriety

As the participants in this study attended AA to recover from alcoholism, they drew from a diversity of personal experiences to construct a life without alcohol. As shown in Fig. 1, structuring of the emotional experiences of study participants who have achieved long-term recovery from alcoholism reveals the emotional attitudes that enable abstaining alcoholics to avoid relapse. According to the analysis depicted in Fig. 1, participants first gain objectivity (1) and then strive to maintain an attitude of acceptance (2). Participants repeated these tasks, set their own goals, and demonstrated devotion to discipline toward one’s goals (3).

As participants incorporated disciplined, spiritual efforts into their daily lives, examined their inner selves, and strove not to depend on alcohol, they recovered their selves. In addition, successful long-term abstinence gave participants confidence and helped them regain their self-esteem. The precepts of a mutual-help group call for humility. Therefore, to avoid being enamored by the success of their long-term abstinence, participants also constantly cautioned themselves regarding their own feelings, words, and actions. These processes fit into Prochaska and DiClemente’s Stages of Change model [26] as follows: gaining objectivity (1) resembles the transition from the Precontemplation stage to the Contemplation stage, an attitude of acceptance (2) resembles the Preparation stage, and devotion to discipline toward one’s goals (3) resembles the Action phase. Because alcoholics are prone to relapse, during the Maintenance phase, they require comprehensive support from their sponsors in AA and must strive daily to avoid stimuli that would destabilize their emotional balance. Therefore, study participants’ processes of change were not direct but instead involved repeated reversion to each stage.

This study’s finding that abstinence is an ongoing process is consistent with that of studies conducted with AA members in Taiwan [27, 28]. In the present study, participants strove to regulate their emotions daily to preserve their emotional balance and prevent relapse. Indeed, as participants’ responses show, these efforts led to fledgling new selves that do not require alcohol. These new selves were conceivably the result of a focus on participants’ emotions and successful long-term abstinence mean duration of 13.76 (range, 1–35) years.

Even if participants had relapsed, their efforts to continue their programs would likely still have led to internal growth and reduced their need for alcohol. Recovering one’s contradictory self (4) showed an emotional conflict in which continued sobriety is impossible without abandoning the narcissistic elements characteristic of abstaining alcoholics and without acquiring objectivity and maturity [25]. All participants said that they were utterly incapable of completing these processes on their own.

“Wanting others to recognize your existence” in “Recovering one’s contradictory self (4)” seemed to stimulate factors that inhibited the progress of emotional processes conducive to involvement with AA and supporting sustainable recovery. Bearing this in mind, as long as the participant practices the AA program in the context of fellowship through AA, he or she may be able to remain sober over the long term. This might even lead to the further idea of “emotional sobriety” [29], a concept advanced by Bill Wilson (Bill W), one of the co-founders of AA. While the importance of the concept of “emotional sobriety” is recognized among some Japanese AA members, it has not yet become popular in Japan. It will be necessary for Japan’s healthcare professionals to continue to watch and understand AA’s future activities, so that they may become a bridge between healthcare organizations and AA, connecting those who are wrestling with drinking problems to AA and continuing to work toward the promotion of AA.

### AA programs that lead to long-term abstinence

The AA was born in Akron, Ohio, on June 10, 1935. Although the sample size of this study was small, its data support the efficacy of the AA program and fellowship through AA in Japan. According to the 12 steps of AA (Table 2), gaining objectivity (1) was conceivably enabled by participants taking stock of their own lives in AA steps 4 and 5. An attitude of acceptance (2) was conceivably enabled by study participants continuing the 12-step program after accepting AA step 1—the most important of the 12 steps, and a difficult one to accept. Devotion to discipline toward one’s goals (3) signifies the continued practice of the entire 12-step program. As can be understood from an examination of Fig. 1, recovering from alcoholism requires tireless effort. Although several studies have indicated that the AA program is effective [30–33], this effectiveness is still being debated due to doubts voiced by some health care professionals [34]. Regarding the fact that the AA program is believed to be empirically effective within the 12-step culture, addiction professionals’ understanding of the culture are said to affect how they support their clients [35]. A deeper understanding of the 12-step culture may be necessary for nursing professionals in Japan.

**Table 2:** The 12 steps of AA

1. We admitted we were powerless over alcohol - that our lives had become unmanageable.	
2. Came to believe that a Power greater than ourselves could restore us to sanity.	
3. Made a decision to turn our will and our lives over to the care of God as we understood Him.	
4. Made a searching and fearless moral inventory of ourselves.	
5. Admitted to God, to ourselves, and to another human being the exact nature of our wrongs.	
6. Were entirely ready to have God remove all these defects of character.	
7. Humbly asked Him to remove our shortcomings.	
8. Made a list of all persons we had harmed, and become willing to make amends to them all.	
9. Made direct amends to such people wherever possible, except when to do so would injure them or others.	
10. Continue to take personal inventory and when we were wrong promptly admitted it.	
11. Sought through prayer and meditation to improve our conscious contact with God as we understood Him, praying only for knowledge of His will for us and the power to carry that out.	
12. Having had a spiritual awakening as the result of these steps, we tried to carry this message to alcoholics, and to practice these principles in all our affairs. Source: Alcoholics Anonymous. New York: Alcoholics Anonymous World Services Inc., 2001^8)^	

### Importance of dealing with emotional issues

In adhering to a 12-step program, participants must overcome the temptation of relapse brought about by unstable post-abstinence emotional balance. As the present study found, the key to overcoming this transition is the challenge to the participant. For alcoholics, the emotional experiences triggered by daily events can easily lead them to drink. Therefore, dealing with such emotions is a crucial task. To maintain emotional balance, alcoholics must acquire individualized coping mechanisms that serve them best. However, in addition to the problem of susceptibility to depression after abstinence, many people who rely on drugs and alcohol are said to have difficulties with emotional intelligence, which constitutes the ability to know, understand, and regulate one’s own emotions [36]. In addition, lower emotional intelligence has been suggested to be associated with additional alcohol- and drug-related problems [37]. Furthermore, alcoholics have long been said to suffer from alexithymia [38, 39], defined as awareness of one’s own emotion, difficulty in verbalizing that emotion, and lack of reflection [40]. Nursing professionals in Japan must support alcoholics emotionally to encourage their recovery [41]. It is also important for nursing and health care professionals to introduce participants to AA, which provides alcoholics with emotional support. Another role that nursing professionals should play is to recognize the activities of AA in their region and build an environment that encourages recovery from alcoholism.

### Limitations

This study is limited in that it involved a small number of Japanese AA members. In the future, it is necessary to conduct research that involves female and elderly AA members to enable health care professionals to have a better understanding of AA.

## Conclusions

This study examined the emotional process of AA members in Japan who are striving for sobriety while attending AA in a country tolerant of drinking. Long-term abstinence was achieved through the following process: 1) gaining objectivity, 2) striving to maintain an attitude of acceptance, and 3) remaining devoted to discipline for one’s goals, thereby 4) recovering one’s contradictory self. This was an iterative process that unfolded with effort over time. Structuring of the emotional experiences of Japanese AA members striving for sobriety revealed that they strove daily to make efforts to maintain sobriety by regulating their emotions. These efforts are thought to have led to spiritual progress and a greatly reduced need for alcohol. The findings indicated that Japanese nursing and health care workers should be willing to learn from AA members, such as by understanding the 12-step culture. The data also suggest the need to create environments conducive to AA activities, from which many alcoholics derive emotional support. The current research serves a step toward broadening the knowledge of health care professionals about AA in diverse cultures.

## Data Availability

The data that support the findings of this study were used under license from the Ethics Committee of Kanazawa University School of Medicine and are not publicly available due to ethical restrictions regarding their sharing. However, the data may be made available by the authors upon reasonable request and with permission from the Ethics Committee of Kanazawa University School of Medicine.

## Appendix

**Table 3:** Interview guide

I would like you to talk to me about	
- Your age, family structure, your job, and history of abstinence	
- How did you feel when you felt the urge to drink alcohol?	
- [How has your sobriety been recently and how will you continue on in life from now?	
・How did you start drinking, why did you need alcohol, how did your mood change after drinking	
・What was the reaction from your family members and other people around you, and how they have changed since you began attending AA meetings?	
・Why and how did you start abstaining from alcohol?	
・When did you become involved with AA?	
- Why are you not drinking alcohol now?	
- Do you ever feel the urge to drink?	
- How do you cope if you want to drink alcohol?	
- What situations do you try to avoid?	
- What do you want to be in the future?	

## Abbreviations

AA: Alcoholics Anonymous
KJ Method: Kawakita Jiro Method
